# Book Reviews

**Published:** 1806-07

**Authors:** 


### CRITICAL ANALYSIS
OF THE
### RECENT PUBLICATIONS
ON THE
### DIFFERENT BRANCHES OF PHYSIC, SURGERY, AND MEDICAL PHILOSOPHY.

---

### *Edinburgh Medical and Surgical Journal.*
Number 6. April 1806.

" Article 1.—*Case of Abscess in the Abdominal Muscles, which terminated fatally.* By CALEB CROWTHER, M. D. Wakefield."

This is a very interesting paper, but leads to no other practical purpose, than to show the difficulty of acquiring a precise knowledge of the history of a disease, particularly in that class of our patients, whose various occupations engage their whole thoughts, and who are not sufficiently aware of the importance attending a scrupulously faithful narrative.

Another Case is subjoined by the author, which appears so extraordinary, that we shall give it in his own words.

" *Case of Syphilitic Ulceration of the Skin, accompanied with Caries of the Tibia.*

" ON the 4th December 1803, I was sent for to visit A. B. æt. 19, whom I found labouring under hectic fever, accompanied with very frequent cough and copious expectoration, the urine being high-coloured, and depositing a copious lateritious sediment; the pulse 120 in a minute. He had great thirst, was very restless, and had profuse nocturnal perspirations. The countenance had a melancholy aspect; the appetite was very bad. On the tibia of the left leg there was an ulcer about one inch and a half long, which had destroyed the periosteum, and rendered the bone carrious. The probe passed readily into the numerous cavities of the denudated bone. From the previous history of the disease, it was evident that this ulcer was of syphilitic origin. For six or eight months prior to the ulceration, a node had made its appearance on the tibia, attended with severe nocturnal pains, which gradually increased in size until the integuments burst. The ulcer had been dressed daily with mild digestive by the surgeon who attended him. He was taking two of the following pills every night at bed-time.

℞. Hydrarg. Calcinat. gr. iij.

P. Opii puri gr. vj.

Confect. Aromat. q. s. ut ft. pil. N° xij.

and and Decoct. C. Cinchon. ℥j. thrice a-day, with a few drops Tinct. Opii and nitric acid.

"I ordered the pills to be continued, a pint of Decoct. Guaiaci, with 40 drops of nitric acid, to be taken, at divided intervals, daily. The ulcer to be dressed with lint soaked in Tinct. Myrrhæ, and a warm linseed poultice. The dressings to be changed thrice a-day.

"*Dec. 8th.* I found my patient this day astonishingly better in every respect. The pulse was natural. The hectic fever and its accompanying symptoms had vanished. The cough and expectoration had nearly ceased. The ulcer put on a healing appearance, and the appetite returned.

"He was ordered to continue his medicines, and, in the course of about a week, was sufficiently recovered to be able to superintend his business. He continued gradually to recover until the 12th January 1804, when the ulcer healed, and he was in perfect health. The leg has since continued sound; and he is, at this day, subject to no complaint, except occasionally to nocturnal pains in the bones.

"This was the most speedy cure of a chronicc disease which I ever recollect to have witnessed; indeed, unless I had myself attended the patient, and seen the rapid recovery, I should have had much difficulty in attaching credit to it. It appears that the lint and the poultice absorbed the purulent matter from the ulcer as it was secreted, in consequence of which the hectic fever ceased. From the carious state of the tibia I fully expected that an exfoliation would have taken place."

The cure was undoubtedly very speedy; but if the bone really was *carious*, it is not a matter of doubt, but of certainty, that exfoliation either has or must hereafter take place. It is however not improbable, that such a process had been completed before Dr. Crowther had seen the patient, which may account for the speedy cure much better, than by supposing that the hectic was subdued from the absorption of the purulent matter by the lint and poultice. We conceived, long before this time, that the theory, which attributes hectic to the absorption of matter, was pretty generally exploded.

"Article 2.—*Remarks on the Internal Use of Tincture of Cantharides in Gleet and Leucorrhœa; illustrated by Cases.* By John Roberton, Surgeon, Edinburgh, and Member of the Royal Medical Society of Edinburgh."

Mr. Roberton's Theory might have been spared: his facts, however, are very important, as is every fact which relates to a frequently troublesome disease. It is well known, that whether the inflammation in these parts is active or passive, if our readers chuse to admit Mr. Roberton's expression, stimulating applications are frequently useful: Witness the use of the solution of hydrargyrus muriatus in virulent gonorrhœa. The truth is, that where where the inflammation is local, the exciting a new action in the part, is often sufficient to supersede the old. We mean, not by this to undervalue Mr. Roberton's communication, but conceive, that the theory he proposes would much lessen the usefulness of many of our more stimulating remedies.

"Article 3.—*Remarks on the Dracunculus, or Guinea Worm, as it appears in the Peninsula of India.* By NINIAN BRUCE, A. M. Surgeon of the 1st Battalion of the 88th Regiment, or Connaught Rangers.

"Article 4.—*Cases of Guinea Worm, with Observations.* By Mr. PATTON, Surgeon of the Cirencester."

These two Articles contain a series of facts, very useful to be recorded in illustration of an obscure disease. They are too long to copy, and of a nature which does not admit abridgement, being principally cases and events which will much assist any future enquiry on this intricate subject.

"Article 5.—*Case of Encysted Ascites, with Hydatids.* By K. MACLEAY, M. D. Oban."

This case is no otherwise remarkable than in the number and size of the hydatids. The whole measured 35 English pints; the size of a great proportion exceeded that of a large orange.

"Article 6.—*Description of the Koutam-Poulli, showing, contrary to the commonly received Opinion, that it does not afford Gum-Gamboge.* In a Letter to Dr. J. ANDERSON, Madras, from Dr. WHITE, Cananore."

Though the author does not pretend to ascertain the precise tree which affords the above substance, yet it certainly is not unimportant to prove, that it cannot be extracted from that to which it is supposed to owe its origin.

"Article 7.—*Observations on secondary Hemorrhage, and on the Ligature of Arteries after Amputation, and other Operations.* By the INQUIRER."

The principal object of this paper is to show the inutility of, and disadvantage attending, broad ligatures for securing arteries. The author shows that the thinnest ligature, drawn ever so tight, is insufficient to cut through an artery; and that since the Alansonian mode of operating, the intention being to unite the covering of the bone by the first intent, we cannot introduce too little extraneous matter. He therefore proposes a ligature of a single silk thread, one end of which should be cut off before the cut surfaces are brought together.—There is certainly nothing objectionable in this plan; but we suspect secondary hemorrhages are induced more by a state of the blood or constitution, which unfits the former for a due coagulation, and the adhesive inflammation, than from any defect in the form of the ligature. We would further remark, that the method proposed by the author, of carefully denuding the artery, has been thought, by some, to produce produce a sloughing of the vessel (above the ligature) by depriving it of those vasa vasorum to which it owes its nourishment. This paper seems to have been suggested by Dr. Jones's ingenious publication.

" Article 8.—*Case of Teeth and Hairs found in the right Ovarium.* By JAMES ANDERSON, Fellow of the Royal College of Surgeons, Edinburgh."

" Article 9.—*The Efficacy of Inoculated Small-Pox in promoting the Population of Great Britain.* By R. GILLUM, M. D. Bath."

" Article 10.—*Essay on the External Use of Oil.* By WILLIAM HUNTER, A. M. Master of the Asiatic Society of Calcutta, and Surgeon of the Marine Establishment, Bengal."

This paper abounds with learning, good sense, modesty, industry, and impartiality. But whilst we admit all this, we are not perfectly satisfied as to the *cui bono* of such a numerous host of quotations. To satisfy our readers on this subject, we shall extract the result of the author's researches and his own remarks.

" From what has been said, we may draw the following conclusions:

" 1. That the application of oils, and other unctuous substances, to the skin, serves to guard the body against the inclemency of the weather, particularly cold and moisture.

" Dr. Sparman says, the ointment of soot and grease, with which the Hottentots smear their bodies, defends them so much from the action of the air, that they require very few clothes. And Dr. Currie, with a similar intention, recommends the use of inunction to Europeans in warm climates, especially after warm bathing, to defend the body from the chilling effect of evaporation. He adduces the example of savage nations, who compensate in this way for the defect of clothing; and observes that the ancients were accustomed to anoint their bodies, before the exercise of swimming, to mitigate the shock of immersion. In the moist climate of Bengal, the practice of inunction is more frequent than in the northern and western parts of Hindostan, where the air is drier.

" 2. It may prevent too profuse perspiration in hot weather, which is one cause of debility. It seems to be with this view that Hippocrates recommends, in summer, the wearing of clothes imbued with oil; and we see that the Asiatic physicians entertain the same idea. Murray is of the same opinion. Dr. Cullen says there is no just foundation for this; but the only argument which he produces against it, viz. " the general practice of the ancients, as well as of Asiatics in modern times," may only prove that a diminution of perspiration, which, though perhaps very considerable, will be compensated by other secretions, is attended with no bad effects on the system. Dr. Currie says, that unguents of a proper consistence may retard excessive sweating, yet not obstruct struct moderate and necessary perspiration; and, being themselves evaporable, they may keep up a coolness that shall diminish the necessity of the natural discharge. The real state of the fact can only be ascertained by statical experiments.

" 3. May it serve as a protection from contagion? Mr. Baldwin thinks that persons so prepared may attend their friends in the plague, without the apprehension of danger. And a similar practice is hinted at by Dr. Monro Drummond. If the practice has any effect in this way, it is by obstructing one channel of contagion, that by the skin. But the experiments of Mr. Seguin and Dr. Currie make it rather improbable that contagion is ever conveyed in this way, while the epidermis is sound. At any rate, it is evident that, without other precautions, this must be unavailing.

" 4. May nourishment be thus conveyed? Dr. Cullen says, the absorption of oils from the surface is never in considerable quantity; and from the experiments related by Dr. Currie, we must conclude that when the body is immersed even in watery fluids, much less is absorbed than had been supposed. The relief to thirst experienced from that practice must therefore be accounted for by the diminution of perspiration, and the sympathy of the vessels of the mouth with those of the skin. It is also worthy of remark, that Alpinus, in his minute account of the process used in Egypt for fattening the human body (an art which, it seems, is there reduced into a system, and of which the bath is a principal instrument) does not mention unction as applied to that purpose.

" 5. It is worthy of trial in the incipient stage of plague, and, combined with internal exhibition, in the bites of venomous animals, of the mad dog, and in tetanus. But we have not yet sufficient grounds of confidence in it to supersede the use of other remedies; and therefore it should be used so as not to interfere with their exhibition.

" 6. Its utility in dropsy appears to be better founded; and as it does not prevent the giving of proper medicines by the mouth, it ought, in general, to be used as a powerful auxiliary. In this as well as the last instance, it must be remembered, that as much is to be expected from the brisk and long-continued friction as from the oil."

" Article 11.—*Case of Crural Hernia, in which the Obturator Artery surrounded the Mouth of the Sac.* By JAMES WARDROP, Fellow of the Royal College of Surgeons, Edinburgh."

In performing so important an operation, it is certainly right to be aware of every possible accident and variety in the disposition of the vessels. We hope no one, who undertakes such an operation, is ignorant of either. Mr. Wardrop suspects, that the distribution he describes, is more common than is usually suspected: This is very probable. But when we reflect, that the whole operation is performed, *manu suspensa*, we are less surprised, if the operator has usually escaped those dangers, which such a state of parts might otherwise have produced.

"*Cow-Pock Inoculation vindicated and recommended from Matters of Fact.* By ROWLAND HILL, A. M. *with the Report of the Medical Council of the Royal Jennerian Society annexed.* 8vo. pp. 72. London, 1806."

This little Work, dedicated to his Excellency the Duke of Bedford, Lord Lieutenant of Ireland, President of the Royal Jennerian Society, &c. &c. &c. by the Rev. Author, himself a Director of the Institution, has afforded us amusement and gratification in its perusal; and we anticipate, with pleasure, that practitioners will, by the public exposure that it affords of the unfair conduct of the opponents of vaccination, be saved much of the trouble which every where falls upon them in removing the fears of those who have perused or heard of their mis-statements. Mr. Hill's Matters of Facts, will, no doubt, from the known benevolence, disinterestedness, and devotedness of his character, outweigh all the unfounded assertions of the adversaries with the public at large. His peculiar manner of mingling the pleasant with the serious, the humorous with the severe, in treating our opponents, we think very happy; and, faithful to that which seems to form the unwearied business of his life, he glances, in the conclusion, with a pious fervour at that Power which does the *sacred* work, and makes us wisely *good.*

It has often been hinted to him, by the most active members of the society, that his writing on the subject might be peculiarly seasonable, as coming from a disinterested individual; the motto on his title, from an arch-opponent's book, seems introduced with a kind of drollery. It would justify a writer of inferior abilities to those of this author in taking up the pen; it would form the highest eulogium ever pronounced on vaccination when compared with the small-pox

"Cow-pox Inoculators may well exclaim against the *cases* of small-pox inoculation,—when their trade is *otium cum dignitate;* and can as well be carried on by a fool as by a philosopher."

"Dr. Mosely."

The reverend and benevolent author has prefaced his pamphlet with a short advertisement, which, with some extracts, must conclude our notice of it.

"It is readily ackowledged that nothing new can be advanced on a subject which has already been so fully investigated, and so ably defended by many gentlemen, of the highest respectability, in the medical profession; yet having been frequently requested to add my testimony to that of others, for the following reasons I readily comply.

"*First.* I have it in my power to speak to matters of fact, perhaps more so than many of the most active among the faculty themselves, having inoculated near Five Thousand subjects with my own hand, exclusive of many more who have been vaccinated between Dr. Walker and myself, at the School Room of this Chapel, under the patronage of the Jennerian Society.

"*Secondly,*

"*Secondly.* It appears that a small publication in a concise and plain stile, and at a low price, containing an abridgement of what has hitherto been published, was greatly needed for the general good.

"*Thirdly.* As a mere hint from the pulpit in different places, has frequently produced some hundred of *customers*, from a kind persuasion that I could have no self-interested motive whatever, I wish to try what next could be done from the press; and as I am told my name has had some influence over the minds and prejudices of many, I am in hopes that I may be able to prevail with others, to resist the stratagems of a few interested and artful men, against such a weight of authority and evidence, as perhaps was never resisted before.

*Surry-Chvpel, March* 25, 1806. R. HILL."

"The advocates for christianity are, of course, the friends of humanity; consequently, every discovery which ameliorates human woe, well deserves the patronage of all those who wish to exemplify the truly christian mind.

"As I aim at brevity, I immediately come to matters of fact, by giving a plain statement of what has fallen within my own knowledge as an inoculator for the public good.

"My first efforts commenced early in the summer of 1804, in and abou Wotton-Underedge, in Glocestershire, where I possess a retired habitation; that neighbourhood being rather populous, I had near twelve hundred applicants. At Hillesley, a village about three miles from that place, the Small-pox began to make its appearance; one person I am told fell a sacrifice to the disease, and another did but just escape with his life; this drove hundreds to me for protection, and the Small-pox was immediately non-existent in those parts.

"Having such ample proof of this beneficial discovery, during the same summer, when on a visit to Portsmouth, to supply for my worthy friend Mr. Griffin, while he was engaged for me at Surry Chapel, in London, I conceived it my duty, and especially as it was by the peculiar desire of Mr. Griffin, to encourage and recommend vaccination in those parts also; unhappily, however, the vaccine matter which I had preserved at Wotton Underedge, having been too much exposed to light and air; which I have since found speedily destroy its active influence on the constitution; I could not raise a single pustule, though I attempted it on many subjects. I was the more concerned at this event, as Mr. Griffin had fully determined to take up the lancet, and to offer vaccination with the utmost freedom to all who might chuse to apply; and I was satisfied that a minister so much beloved by his numerous congregation, and so deservedly respected by the public at large, might gain an ascendancy over peoples' prejudices, much for the public good, and for the preservation of the lives of hundreds of the infant race.

"Upon Mr. Griffin's return, he not only administered vaccination to many applicants, but has moreover favoured the public with a treatise so full of information, containing such a variety of incontestible facts in favour of vaccination, as must convince every unprejudiced mind of the perfect security, and extensive utility, of this very blessed discovery, for the comfort and protection of millions of the human race.

" In the autumn of the same year, while on a visit to Bristol, I found I had there also sufficient interest to prevail with hundreds to forego their prejudices against vaccination; and to submit to be protected from the dangerous ravages of the Small-pox, which are known to be so exceeding fatal in all cities that are large, populous, and close: this visit, which I repeated on the autumn of last year, when many more were added to the list of those which were inoculated before; at the same time I made diligent enquiry respecting the *mischievous* effects of my proceedings in the former year; but without a single discovery that could create the least possible suspicion and regret; numbers of the poor people from the Kingswood Forest, near that city, came by groupes for inoculation.

" On my return to London, some people from Clapham requested my assistance, to secure their offspring from the ravages of the Small-pox, which began to threaten considerable havoc in that populous village: here also many prejudices were excited, because one person was said to have sustained an attack from the Small-pox after vaccination; for the sake of argument it shall be granted, but after having vaccinated near eighty with my own hand, which was followed by a publication delivered in the church, that the medical gentlemen would administer vaccination, gratis throughout the parish, the ravages of the Small-pox were completely at an end; nor can I find that either itch, or mange, or Cow-pock ulcers, or any other terrible calamity that has put the brains of the redoutable Dr. Mosely and his colleagues into such a tremendous ferment, has appeared to torment a single individual among them all.

" I pass over some less exertions in the same cause, and return to my procedure in the spring, 1805.

" On a visit to Chatham, I had speedy information that the ravages of the Small-pox began to be very deplorable through Chatham, Rochester, and Stroud; being three towns, though containing but one street; extending upwards of two miles in length, together with crouded courts and other contiguous places, rendering the population the most numerous of any town in Kent.

" By the assistance of my worthy friend, Colonel Burn, of the Royal marines, I immediately procured two recruits of that corps; for, notwithstanding these few antivaccinarian doctors have proved that this strange rage for inoculation is *folly* and *madness;* yet still such *fools* and *madmen* are found to command the army, as that no young recruits are allowed to enter the barracks racks but as they are vaccinated immediately, if it appears they have not had the Small-pox before they enlisted into the service.

"With these two recruits I immediately set to work, and having appointed the people to meet me at three different places of worship, as being the most commodious for my purpose, in the three towns I inoculated three hundred and twenty subjects within two days.

"Not only at Chatham, but at Sheerness also, many have been persuaded to seek for the protection that vaccination affords: but as I had nothing but dry matter, I fear that very few were properly infected thereby; however, having shewn Mr. Prior, the minister, and others, how to apply the lancet, and having described to them the nature of the pustule and the effects of the slight disease, I am in great hopes that the blessing of vaccination will be established in those parts.

"During the course of the summer, I paid a visit to my friend Mr. Sibree, of Froome. Being the minister of a large congregation in that populous town, he particularly requested me to recommend the blessing of vaccination to the public notice, especially as the Small-pox had been very fatal about those parts. At Shepton Mallet, about ten miles from thence, not less than seventy had fallen victims to that disease, when, if it had not been for ignorance, covetousness, or art, all of them might have been preserved.

"I recommended from the pulpit vaccination; the concourse which attended on Monday, pleased and surprized me not a little; in the course of two hours I inoculated not less than two hundred. Mr. Sibree also, who came in late from the country, after the business was nearly finished, applied the lancet to about twenty more. This will not be a cause of surprise, when I state that the overseers of the parish asked my leave to send the bellman round, announcing my willingness to vaccinate every person that chose to apply. Immediately after this, I received from Mr. Priestly, the minister of Shepton Mallet, a pressing invitation to encourage vaccination at that town also, where people had been such sufferers from the Small-pox. This I considered a call of humanity, and immediately obeyed.

"*Extract from Mr. Sibree's Letter.*

"I examined nearly all the children and persons you inoculated at Froome: all had taken, all did well; no sore arms, or scabby effects produced. I still go on; had ten patients last Thursday, expect double the number next Thursday. I have inoculated near 500— all have done well.

Yours, &c.

*Froome, Ap.* 1, 1806. JOHN SIBREE."

"I have nothing further to relate to the reader, as it respects my practice as a vaccine inoculator for the public good, than to give a concise account of some very successful efforts during my last summer's excursion into Wales.

"While in Pembrokeshire, I inoculated nine hundred ninety-six, and having taught others the use of the lancet for the same beneficial purpose, I availed myself of their assistance, and consequently may add, I believe, another hundred to that list, making up eleven hundred in the whole; thus the poor people in and about the neighbourhood of Haverfordwest, being one of the most populous parts in Wales, are mercifully secured from a contagious disease, which would very probably have soon made its inroads among them; while no less than three hundred in the town of Carmarthen, at the distance of about 30 miles from Haverfordwest, were carried to the grave the victims of that awful plague.

"When I first began this little work, Dr. Rowley was then a living opponent; his book, however, yet lives to promote the destruction of multitudes, though he himself lives no more: as for Dr. Moseley's pamphlet, one would have thought it must soon have destroyed itself.

"Having inoculated in different places, not less than 4840 subjects, independent of 3720 and upwards, which have been inoculated at Surry Chapel School-Room; I have not, as yet, met with ONE SINGLE FAILURE, though upon the repetitions of my visits, I have at all times made it a point to enquire, with the utmost diligence in my power; nor yet in any one point of view, have I seen any of those distresful consequences that have been brought forward with so much *art* and *downright falsehood*, to alarm the fears and terrify the imaginations of the public.

"It is in vain to expatiate against the artful stratagems, the self-interested motives of a Moseley, a Birch, a Squirrell, and others of their antivaccinarian satellites, who can dance around the destructive altar of variolous inoculation, by their wanton pretensions to sportive wit; while the lives of thousands are at stake. It is in vain to tell the public that the characters of these men, as it respects their attempts against vaccination, are gone, for ever gone; in having been detected, that either through ignorance or art they have been guilty of printing and reprinting a set of tales which never existed, or exaggerated statements which they must know or might have known, were nothing to the purpose, while the cause of all the fatal mischief must rest with them alone.

"Notwithstanding the prejudices of the public against all new discoveries, the blessing of vaccination for several weeks on the average, had reduced the deaths by the Small-pox to about Ten per week; after these men and their adherents had been making their clamours, by posting up their public hand-bills, by advertising in all the public papers, and other periodical publications, they soon amounted to one hundred per week; while the number of applicants for vaccination were universally diminished."

---

### *Dr. Jones's Treatise on Hæmorrhage.*

[ Concluded from our laſt Number, p. 483. ]

We left this intereſting work, ſtrongly impreſſed with the importance of the author's experiments, the faithfulneſs of his relations, and the candour with which he treats others. We ventured indeed to ſuggeſt, that in the curative and preſervative proceſſes he ſeemed to us to impute too little to original laws of the conſtitution, by which certain actions follow certain ſtimuli.

The ſecond chapter is on the means which nature employs for ſuppreſſing hæmorrhage from punctured or partially divided arteries, and on the proceſs of ſeparation which takes place in thoſe arteries. Theſe experiments are ſtill more intereſting than the foregoing, and the candour and ingenuity of the writer not leſs conſpicuous. By the reſult of the whole it appears, that when an artery is punctured longitudinally it heals as perfectly as any other clean inciſion, inſomuch that even the cicatrix in a ſhort time cannot be diſcovered; that the difficulty of healing is in proportion as the puncture is more approaching to the tranſverſe figure of the veſſel; that if a tranſverſe or oblique inciſion is extended along leſs than one-fourth of the diameter of an artery, the wound from the contraction of the veſſel approaches more to a circular form, and that the next reſource of nature is to fill up the breach by coagulated lymph; ſtill, however, preſerving the cavity of the artery, though imperfectly, as the lymph within ſomewhat encroaches upon it. The author found that the coagulum has on theſe occaſions become vaſcular over the artery, ſo that a proviſion was evidently made for its complete repair, after which, had the animal been permitted to live, ſuperfluous coagulum probably would have been abſorbed.

But if the inciſion was made in an oblique or tranſverſe direction of an artery, ſo that more than one-fourth of its diameter has been divided, the circular aperture becomes ſo large, and probably the reſtoration of its arterial function ſo difficult or ſo remote, that a new action muſt now be ſet up; that is, the preſervation of the artery and its functions being no longer within the power of the conſtitution, the canal of the artery is deſtroyed altogether. Ulceration takes place ſo as to divide the whole diameter of the veſſel; after which nothing remains to prevent the retraction of the artery, and the ſame proceſs follows as in other inſtances of divided arteries.

This chapter concludes with ſome enquiries, why ſpurious aneuriſms in the human ſubject might not be remedied in a manner ſimilar to what was experimentally proved in brutes. As the author ſeems to admit that the difference is not eaſily accounted for, we ſhall certainly not undertake it. We heartily approve of his propoſed plan to accompliſh the natural cure in the human ſubject, if that can be done. But we know not why he ſhould be ſo fearful of being ſuſpected of "laying down this as the proper treatment of a wounded artery." "On the contrary," ſays he, "I am convinced

 that that in every cafe in which *it can be done*, it is beft to tie the artery above and below the wounded part, and to divide it completely between the ligatures." In our opinion the author would have done better to have faid where it *muft be done*. We are ready to give him due credit for his attention to Celfus, to Petit, and to Murray; but we think, after his experiments he may be allowed to judge for himfelf. We are aware alfo of the "difficulty which muft attend an attempt to compress a wounded artery, fo as to keep its lips in contact, and at the fame time neither obliterate its canal, obftruct the circulation of the blood through the limb, nor affect the return of the blood through the veins fo as to produce tumefaction, and the well-known train of evils thence enfuing." But it muft be remarked, that the obliteration alfo takes place when the artery is tied above and below the puncture; and not only have we this inconvenience to ftruggle with, but an open fore whofe healing is interrupted by a double ligature. Add to this, the operation is by no means a light one if the parts are much obfcured by blood, and, as we cannot with any certainty afcertain under fuch circumftances, whether ulceration has already commenced in the artery, fo we muft always remain in fome doubts as to the fuccefs of our operation. It is unneceffary to add, that fome cafes, conducted by very able furgeons, with every poffible advantage, in our own days, have proved unfuccefsful. We are ftill ready to admit the improbability of producing the healing of an artery which has been kept fome time divided, but we may at any time attempt to imitate part of the procefs of nature in obliterating the cavity of an artery, and thus even without the neceffity of ligature or ulceration to deftroy its continuity.

The third chapter leads moft of all to thofe practical advantages which are derivable from the foregoing experiments. It is fuperfcribed —"On the Operation of the Ligature, fhewing that its immediate Effects is to divide the middle and internal Coats of an Artery, which gives rife to the adhefive Inflammation."

Our author's experiments on this occafion are fo complete as to filence every cavil. If therefore we differ from him, it cannot be in the refult of what he faw, the inference he draws, or even the practical advantages he propofes. But as we find hæmorrhages are for the moft part prevented with ligatures of all defcriptions, and that under the management of the moft careful furgeons, and of practitioners who operate on different principles, they ftill fometimes occur, we may be allowed to queftion fome of the caufes of fecondary hæmorrhage produced by our author, as well as the means he propofes of preventing them. At the fame time we fhall, in juftice to his ingenuity and labour, add, that we are obliged to his refearches for the very objections we offer with much diffidence.

Dr. Jones found, that whenever by the force of his ligature he completely divided the middle and internal coats of an artery, it always became impervious, even if his ligatures were removed fo quick that the artery for a time recovered its permeability. This very properly induces him to urge, that all ligatures fhould be regularly rounded, rounded, not too thick, and always drawn ſufficiently tight to cut through thoſe coats. He at the ſame time ſhows, that there can be no danger of cutting through the outer coat, or of producing ulceration of the whole veſſel before adheſion has taken place, for that the adheſion, though produced by coagulated lymph thrown out at the cut extremities of the internal coats, takes place ſomewhat above the ligature. All this he imputes to inflammation, the conſequence of which, is the effuſion of lymph, or what Mr. Hunter calls the adheſive inflammation. But in order to reconcile the various events which he traces in his own experiments, and in thoſe recorded by others, ſome other reſources of nature ſeem to us requiſite. If an artery, after its internal coats are completely divided, remains ſo far permeable that the blood flows without interruption, it is not eaſy to account for its future obliteration by the mere effuſion of coagulable lymph from inflammation. Muſt not the conſtant flow of blood prevent the deſtruction of the canal even ſhould the coagulum become vaſcular. Under ſuch circumſtances it may remain adhering to the ſides of the artery, but we can form no idea of its conſolidation with ſuch rapidity as to interrupt the current of blood after it has recovered its free paſſage. In anſwer to this it may be urged, that the author adduces his experiments. By theſe experiments it appears that the ligatures were applied tight enough to cut through the internal and middle coats of an artery, that they were inſtantly afterward withdrawn by means of a piece twice placed longitudinally with the artery, and included within the ligature. Care was taken to aſcertain that the blood afterwards flowed freely, or as the author expreſſes it, that the *circulation* was perfectly reſtored. Yet when the animals were killed, three days afterwards, the artery was found impervious, adheſion having taken place at the cut edges of the internal coats, and a conſiderable part of the cavity being filled with coagulum.

The author's intention in theſe experiments was to ſhow that the retraction of the artery is not neceſſary for the prevention of ſecondary hæmorrhage, and this he proves beyond all doubt. But when we conſider that ſome of theſe experiments were made on the carotid arteries of a horſe, the force of the blood through which we need not inſiſt on, we cannot help looking for ſome other cauſe for the obliteration of the cavity, beſides the effuſion of lymph from the divided coats. When two coats of an artery are divided in any part of its whole diameter, may we not expect, that the veſſel itſelf, the blood, and the ſurrounding parts, will be impreſſed with the ſame ſtimulus as if the artery were completely divided throughout its whole ſubſtance. In this laſt caſe we find coagulation take place both within and without the artery, and an effuſion of lymph, in order to produce a complete obliteration of the veſſel from the cut part to the next anaſtomoſing branch. That ſuch was the caſe in the preſent inſtance we have reaſon to believe, becauſe the ſame uniform ſeries of actions was traced in every partial diviſion of the internal and middle coats, as were found when an artery was partially ally divided in any but a longitudinal direction throughout its whole fubftance. For if the internal and middle coats were partially divided, the adhefion of effufed lymph only took place on thofe parts. In the experiment related from Poiteau, it appears to us that the fecondary hæmorrhage arofe from the very caufe our author has detected in another place, that is, that the internal and middle coats of the artery were only partially divided; in confequence of this, the fame effect was produced as arifes from a tranfverfe or oblique wound in an artery, that is, ulceration took place in order to complete the divifion and affift the retraction of the artery, by which the extremity might be more readily enclofed in coagulated blood. But before the procefs could be completed, hæmorrhage took place, and the animal died. It appears as if the external wound had remained open, and probably the retraction of the veffel too might have been interrupted by the ligature.

While we make this remark on Dr. Jones's experiments, we are anxious once more to exprefs our acknowledgment to him for his great diligence, fidelity, and the good fenfe with which he conducted his enquiries, and reafons upon them. If we differ from him in the latter, it is becaufe we cannot clearly comprehend that the caufes he affigns are equal to the effects produced, without admitting greater refources in the animal œconomy for the reparation of injuries than he feems to require.

